# Optogenetic Monitoring of the Glutathione Redox State in Engineered Human Myocardium

**DOI:** 10.3389/fphys.2019.00272

**Published:** 2019-04-04

**Authors:** Irina Trautsch, Eriona Heta, Poh Loong Soong, Elif Levent, Viacheslav O. Nikolaev, Ivan Bogeski, Dörthe M. Katschinski, Manuel Mayr, Wolfram-Hubertus Zimmermann

**Affiliations:** ^1^Institute of Pharmacology & Toxicology, University Medical Center Göttingen, Göttingen, Germany; ^2^DZHK (German Center for Cardiovascular Research), Partner Site Göttingen, Göttingen, Germany; ^3^Institute for Experimental Cardiovascular Research, University Medical Center Hamburg-Eppendorf, Hamburg, Germany; ^4^DZHK (German Center for Cardiovascular Research), Partner Site Hamburg/Kiel/Lübeck, Hamburg, Germany; ^5^Institute for Cardiovascular Physiology, University Medical Center Göttingen, Göttingen, Germany; ^6^King’s British Heart Foundation Centre, King’s College London, London, United Kingdom

**Keywords:** optogenetics, engineered human myocardium, redox-reporters, stem cells, cardiomyocytes, fibroblasts, roGFP, GSH

## Abstract

Redox signaling affects all aspects of cardiac function and homeostasis. With the development of genetically encoded fluorescent redox sensors, novel tools for the optogenetic investigation of redox signaling have emerged. Here, we sought to develop a human heart muscle model for in-tissue imaging of redox alterations. For this, we made use of (1) the genetically-encoded Grx1-roGFP2 sensor, which reports changes in cellular glutathione redox status (GSH/GSSG), (2) human embryonic stem cells (HES2), and (3) the engineered heart muscle (EHM) technology. We first generated HES2 lines expressing Grx1-roGFP2 in cytosol or mitochondria compartments by TALEN-guided genomic integration. Grx1-roGFP2 sensor localization and function was verified by fluorescence imaging. Grx1-roGFP2 HES2 were then subjected to directed differentiation to obtain high purity cardiomyocyte populations. Despite being able to report glutathione redox potential from cytosol and mitochondria, we observed dysfunctional sarcomerogenesis in Grx1-roGFP2 expressing cardiomyocytes. Conversely, lentiviral transduction of Grx1-roGFP2 in already differentiated HES2-cardiomyocytes and human foreskin fibroblast was possible, without compromising cell function as determined in EHM from defined Grx1-roGFP2-expressing cardiomyocyte and fibroblast populations. Finally, cell-type specific GSH/GSSG imaging was demonstrated in EHM. Collectively, our observations suggests a crucial role for redox signaling in cardiomyocyte differentiation and provide a solution as to how this apparent limitation can be overcome to enable cell-type specific GSH/GSSG imaging in a human heart muscle context.

## Introduction

Reactive oxygen species (ROS) can be damaging to cells if produced in excess, but also contribute to physiological signaling within and between cells (Sies et al., 2017). In cardiomyocytes, ROS are implicated in fundamental mechanisms of electromechanical coupling, such as the regulation of calcium release from the sarcoplasmic reticulum via mechanosensitive microtubule-dependent oxidation (termed X-ROS) of the ryanodine receptor (RyR2; Prosser et al., 2011) or oxidation mediated homodimer formation of PKGIα, leading to phospholamban (PLN) phosphorylation and enhanced diastolic relaxation (Scotcher et al., 2016). Redox signals have also been shown to alter cardiac metabolism via O-GlcNAcylation of fatty acid transporters (Nabeebaccus et al., 2017). Many other mechanisms have been reported and are summarized in excellent reviews (Burgoyne et al., 2012; Shao et al., 2012; Santos et al., 2016).

Reactive oxygen species are produced by two major mechanisms: (1) as byproduct of the electron transport chain function in mitochondria (Bertero and Maack, 2018) and (2) via the catalytic activity of NADPH oxidases (NOXs; Zhang et al., 2013). The main NOX isoforms in cardiomyocytes are the highly regulated NOX2 and NOX4, which is thought to be constitutively active. Activity of NOX5 is postulated to contribute to redox signaling in cardiac fibroblasts (Zhang et al., 2013), demonstrating cell type specific ROS production in the heart and suggesting cell type specific ROS signaling. Given the short half-life and fast removal by antioxidant enzymes (Stone and Yang, 2006) it is likely that ROS signaling is strictly compartmentalized, as recently reported for X-ROS signaling (Prosser et al., 2011). The identification of the pathophysiological relevance of ROS production in cellular compartments should be facilitated by the use of cell-type and organelle restricted genetically engineered redox sensors (Morgan et al., 2011; Gibhardt et al., 2016). In contrast to classical dyes for ROS quantification - such as dichlorodihydrofluorescein diacetate (H2DCFDA), Amplex^®^ Red or boronate based probes (Rezende et al., 2018) - transgenic sensors allow for stable, long-term measurements of ROS, such as H_2_O_2_ (Belousov et al., 2006; Gutscher et al., 2009; Morgan et al., 2016) and the redox potential of ROS scavengers, such as glutathione (Gutscher et al., 2008).

The widely used glutathione redox potential sensor Grx1-roGFP2 was developed by the fusion of redox sensitive roGFP2 with the human glutaredoxin-1 (Grx1) domain to improve the specificity of roGFP2 toward reporting the glutathione redox state (Gutscher et al., 2008). The sensor exhibits excitation maxima at 408 nm for oxidized Grx1-roGFP2 and 488 nm for reduced Grx1-roGFP2, with emission at 500–530 nm. Ratiometric imaging of the fluorescence emission at the indicated excitation wavelengths allows for the calculation of the ratio of reduced to oxidized glutathione (GSH/GSSG). As the reaction is fully reversible, dynamics of GSH/GSSG changes can be detected in living cells. From this data, the glutathione redox potential (E_GSH_) can be calculated (Meyer and Dick, 2010). Furthermore, localization to cellular compartments can be achieved by fusing Grx1-roGFP2 to organelle targeting sequences, such as in the mitochondria targeted mito-Grx1-roGFP2 variant. Cardiomyocyte specific expression of cytosolic and mitochondrial Grx1-roGFP2 in a mouse model recently revealed that the E_GSH_ in the mitochondrial matrix is more reduced compared to the cytosolic E_GSH_ (Swain et al., 2016). A recent study employed fluorescent redox sensors in a model of sudden cardiac death in guinea pig, elucidating the effect of mitochondrial ROS production in chronic beta adrenergic stimulation (Dey et al., 2018), while others have used organelle targeted redox sensors to investigate the redox effect of insulin signaling in mice (Steinhorn et al., 2017). These studies and their findings demonstrate how redox sensors can add to our understanding in disease initiation and progression. However, they are limited by the use of animal models, with their model intrinsic differences in pathophysiology compared to the human.

Here we report the to our knowledge first application of Grx1-roGFP2 in human embryonic stem cell-derived cardiomyocytes as well as engineered human myocardium (EHM; Tiburcy et al., 2017) for in-tissue glutathione redox state imaging.

## Materials and Methods

### Cloning of Redox Sensor Expression Constructs

Grx1-roGFP2 and mito-Grx1-roGFP2 coding sequences were amplified by PCR from pLPCX backbone vectors (kind gift by Tobias Dick, Heidelberg) with engineered SalI and PacI restriction sites. Sensor sequences were inserted into the multiple cloning site (MCS) of a modified pAAVS-CAG-MCS-EF1-puro vector. Similarly, the coding sequence for Grx1-roGFP2 was cloned into a lentiviral backbone vector (pGIPZ) under the control of the CMV promoter. Correct insert integration was verified by colony PCR, restriction digest, and sequencing.

### TSA Cell Culture

TSA201 cells (human embryonic kidney cells [*aka* HEK293]; ECACC), used to validate transgene expression and localization, were grown in high glucose DMEM (Thermo Scientific) supplemented with 10% fetal calf serum (FCS). Cells were digested using 0.05% Trypsin-EDTA (Thermo Scientific), counted and seeded at 0.05 × 10^6^ cells /cm^2^. Transfection with 2 μg DNA per 6-well for redox sensor integration was carried out using PolyFect Transfection reagent (Qiagen). Cells were selected with 3 μg/ml puromycin.

### Human Embryonic Stem Cell Culture (HES)

HES2 cells (ES International, Singapore) were cultured on irradiated feeders in Knock-out DMEM supplemented with 20% Knock-out Serum Replacement, 2 mmol/L glutamine, 1% non-essential amino acids (NEAA), 100 U/mL penicillin, and 100 μg/mL streptomycin (all Thermo Scientific) and further supplemented with 10 ng/mL FGF2 (PeproTech). For feeder-free culture and differentiation, cells were plated on 1:120 growth factor reduced Matrigel^TM^ (Corning)-coated plates and grown in E8 medium (Stem Cell Technologies). Ethical approval for the use of HES was obtained from the Central Ethic Committee for Stem Cell Research (ZES; permit #12; reference number: 1710-79-1-4-16).

### HES Electroporation

HES2 cells were dispersed into single cell suspensions by incubation with TrypLE (Thermo Scientific). Electroporation with the Neon transfection system (Thermo Scientific) was carried out according to manufacturer’s protocol. Briefly, one million HES2 were resuspended in 100 μl buffer R and mixed with 444 ng pAAVS1-[mito-]Grx1-roGFP2, 28 ng pTALEN-HA-R and 28 ng pTALEN-HA-L (AAVS1 TALE-Nuclease Kit, System Biosciences). Electroporation was performed with a single pulse at 1,200 V for 40 ms. Cells were then immediately transferred onto γ-irradiated feeder plates in HES medium with 5 μmol/L ROCK inhibitor Y27632 (Stemgent). After 4 days of recovery, cells were transferred onto Matrigel^TM^ coated plates and cultured in E8 medium. Selection with 0.4 μg/mL puromycin was performed for 5 days. Single fluorescent colonies were transferred manually and expanded. Genotyping was performed to confirm transgene integration. We obtained one positive Grx1-roGFP2 clone and 14 clones positive for mito-Grx1-roGFP2 of which one clone was selected for use in all further experiments. Pluripotency was confirmed using flow cytometry detection for Oct4 (96 ± 1%, 96 ± 0.5%), Nanog (94 ± 3%, 93 ± 2%) and Tra1-60 (96 ± 1%, 97 ± 2% for the Grx1-roGFP2 and mito-Grx1-roGFP2 lines, respectively, *n* = 3 independent passage; Supplementary Figure 1). Transgene integration was analyzed using Universal Genome Walker kit (Clontech). We could not confirm targeted integration into the AAVS1 locus, but determined random integration into several loci, including chromosome 2, p21, promotor flank region (mito-Grx1-roGFP2) and chromosome 16, STXB1, intron 1 (Grx1-roGFP2).

### Lentivirus Production and Transduction

TSA201 cells were transfected with the Grx1-roGFP2 encoding expression plasmid pGIPZ-CMV-Grx1-roGFP2, packaging plasmid psPAX2 (Addgene plasmid #12260) and envelope plasmid pMD2.G (Addgene plasmid #12259; both kindly provided by the Trono Lab, EPFL) using PolyFect (Qiagen). Culture supernatant containing viral particles was harvested after 72 h and filtered through 0.45 μm filter (Millex^®^ Syringe filter units, Merck Millipore). Cardiomyocytes differentiated from HES2 (hCM) and human foreskin fibroblasts (hFF; SCRC 1041, ATCC) were transduced in the presence of polybrene (0.8 mg/mL, Sigma) and incubated for 72 h at 5% CO2 and 37°C before analysis of transduction efficiency.

### Cardiac Differentiation and Cardiomyocyte Culture

HES2 cardiac differentiation was carried out as previously described (Tiburcy et al., 2017). HES-derived hCM were cultured on Matrigel^TM^-coated plates in RPMI 1640 supplemented with GlutaMAX, 1 mmol/L sodium pyruvate, 100 U/ml penicillin, 100 μg/ml streptomycin (all Thermo Scientific), 200 μmol/L L-ascorbic acid-2-phosphate sesquimagnesium salt hydrate (Sigma-Aldrich) and 2% B27 supplement (Thermo Scientific).

### Engineered Human Myocardium

Engineered human myocardium (EHM) was constructed as previously described (Tiburcy et al., 2017). Briefly, hCM and hFFs were mixed at a 70:30% ratio with pH-neutralized collagen type I and concentrated culture medium on ice (total cell number per EHM: 1.45 × 10^6^). The reconstitution mix was cast into circular molds (450 μL / EHM). After 3 days in culture, consolidated EHM were transferred on stretch devices and cultured for 20 days in IMDM with 4% B27 minus Insulin, 1% non-essential amino acids, 100 U/ml penicillin, 100 μg/ml streptomycin (all Thermo Scientific), 100 ng/μl IGF-1, 10 ng/μl FGF-2, 5 ng/μl VEGF (all PeproTech); supplemented with 5 ng/μl TGF-beta (PeproTech) for the first 72 h of culture. After 21 days of culture, EHM were subjected to isometric force measurements as previously described (Tiburcy et al., 2017) and redox imaging.

### Life Cell Imaging of Mitochondria

Cells were seeded onto glass bottom imaging dishes (Zell-Kontakt) using growth conditions stated above. Staining with TMRM was carried out according to manufacturer’s recommendations. Briefly, cells were incubated for 30 min in a standard cell culture incubator with 50 nmol/L TMRM and 10 μg/mL Hoechst-3342 in normal growth medium followed by two washes with PBS (Thermo Scientific). Cells were then imaged in imaging buffer (in mmol/L: 114 NaCl, 5.4 KCl, 1 MgCl_2_, 2 CaCl_2_, 10 HEPES; pH 7.4) on an Olympus IX81 fluorescence microscope using Xcellence pro software.

### GSH/GSSG Imaging

Transgenic cells were seeded on 25 mm diameter glass coverslips using growth conditions stated above. GSH/GSSG imaging was performed using a pH-buffered imaging solution (in mmol/L: 114 NaCl, 5.4 KCl, 1 MgCl_2_, 2 CaCl_2_ [1 CaCl_2_ for hCM], 10 HEPES; pH 7.4) in a 37°C climate chamber on a Zeiss D1 Observer microscope using ZEN image processing software (Zeiss). Images were acquired sequentially at 400 nm and 505 nm excitation using a YFP filter at 40× magnification every 3 s. Hydrogen peroxide (H_2_O_2,_ a strong oxidant) or Dithiothreitol (DTT, a cell permeable thiol reducing agent) were added after a stable baseline was reached at 30 s. Likewise, lentivirally transduced hCM and hFF were seeded on Matrigel^TM^-coated 24-well imaging plates (ZellKontakt) and fluorescence emission at 510 nm (upon excitation at 405 and 488 nm) was captured with an Olympus IX83 fluorescence microscope equipped with a cellVivo (Pecon) 37°C climate chamber using Visiview Software. H_2_O_2_ or DTT were added after a stable baseline was reached in both cell types. Image analysis was carried out using Fiji-ImageJ with the BioVoxxel Toolbox, Python 3.6 and GraphPad prism 7. ROIs were drawn by hand based on bright field and GFP images to correspond to single cells, as well as a background ROI not containing any cell per slide. Fluorescence intensities for each ROI in each channel were extracted and corresponding background values subtracted. The ratio of the corrected intensity values was normalized to the average of the baseline. Calculation of E_GSH_ was performed as previously described (Meyer and Dick, 2010).

### Mitochondrial Isolation and Western Blot

Mitochondria were isolated from cells as previously established (Acín-Pérez et al., 2008). Briefly, 30 × 10^6^ cells were resuspended, washed with PBS, and the cell pellet was frozen at -80°C and thawed to disrupt the membranes. After thawing, cells were resuspended in 83 mmol/L sucrose, 10 mmol/L HEPES, pH 7.2 and homogenized by 30 strokes using pestle B in a 2 mL Dounce homogenizer. An equal volume of 250 mmol/L sucrose, 30 mmol/L HEPES, pH 7.4 containing complete mini protease inhibitor cocktail (Roche) was added, followed by centrifugation for 5 min at 1,000 g and 4°C to pellet nuclei and remaining intact cells. The supernatant was then subjected to another round of centrifugation for 15 min at 12,000 g and 4°C to pellet mitochondria. The cytosolic fraction in the supernatant was collected for later analysis and the mitochondrial pellet was resuspended in 320 mmol/L sucrose, 1 mmol/L EDTA, 10 mmol/L TRIS–HCl (pH 7.4) containing complete mini protease inhibitor cocktail. Protein concentration was determined by Bradford assay. 25 μg of protein per sample was loaded onto a denaturing SDS–PAGE and subjected to electrophoretic separation at 120 V for 1 h. Proteins were transferred by semi-dry blotting onto a PVDF membrane. Unspecific binding was blocked by incubation with TBS-T containing 5% whole milk powder for 1 h. Primary antibody incubation was carried out overnight at 4°C. After 1 h incubation with secondary antibody solution, membranes were imaged using the femtoLUCENT PLUS-HRP Kit (G-Biosciences) in a Qiagen ChemiDoc^TM^ imaging chamber. For detailed information on the antibodies and dilutions used refer to Table 1.

**Table 1:** Antibodies used for Western Blotting.

Antibody	Species	Supplier	Cat#	Dilution
eGFP Tag	rb	Thermo Fisher	CAB4211	1:1,000
Total OXPHOS rodent WB antibody cocktail	ms	Abcam	ab110413	1:250
GAPDH	ms	ZYTOMED Systems	RGM2-6C5	1:50,000
α-tubulin	ms	Sigma	T5168	1:2,000
Anti mouse HRP	goat	Dako	P0260	1:10,000
Anti rabbit HRP	goat	Dako	P0448	1:5,000

### Flow Cytometry

To assess pluripotency of HES lines, formaldehyde fixed cell samples were stained with Oct4-Alexa-647 (1:50; BD Biosciences), Nanog-Alexa-547 (1:25; Miltenyi), or Tra1-60-Alexa-647 (1:50; BD Biosciences) for 45 min. All samples, including negative controls, were exposed to Hoechst-3342 for nuclei labeling (Thermo Scientific). For a quantitative analysis of transgenic cardiomyocytes, samples were stained with anti-human alpha-actinin mouse monoclonal antibody (1:4,000; Sigma) for 60 min followed by anti-mouse IgG Alexa-633 (1:1,000; Invitrogen) and Hoechst-3342 staining for 45 min. For each sample, a negative control was incubated only with secondary antibody and Hoechst-3342. To assess the transduction efficiency of cardiomyocytes and fibroblasts, transduced and non-transduced cells were stained with SYTOX Red Dead Cell Stain (Thermo Scientific) for 15 min at room temperature and live cells analyzed for GFP expression. All flow cytometry samples were analyzed using a LSRII cytometer (BD Bioscience) and flowing software v2. In fixed samples, gating for intact cells was performed based on FSC-A vs. SSC-A and the Hoechst-3342 signal was used for cell DNA content assessment and exclusion of doublets. Marker gating was performed based on non-stained controls, gating for GFP signal was based on non-GFP control cell lines.

### Statistics

All data are displayed as means ± standard error of the mean (SEM). Biological replicates (n) are indicated with each data set. Statistical tests were performed as indicated in the legends and the main body of the manuscript. A *p*-value < 0.05 was considered statistically significant. Statistical testing was performed using GraphPad Prism 7.

## Results

### Compartment Specific Expression of GSH Redox Sensors

**Figure 1:**
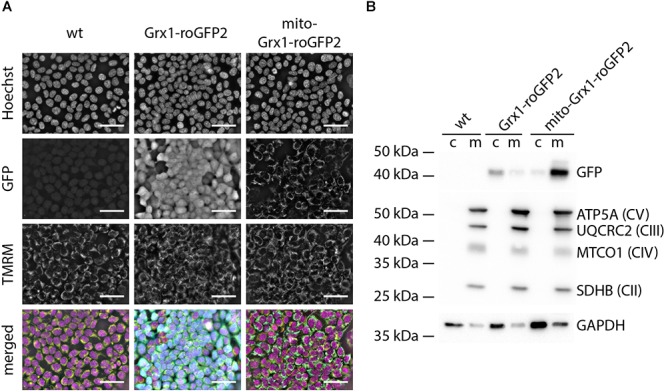
TSA cells express Grx1-roGFP2 in a compartment specific manner. **(A)** Live cell imaging of roGFP-reporter (GFP), TMRM (mitochondria) and Hoechst (DNA) in TSA cells demonstrated the anticipated cytosolic and mitochondrial localization of Grx1-roGFP2 and mito-Grx1-roGFP2, respectively; note that the GFP signal in the Grx1-roGFP2 condition fills the whole cell, whereas the mito-Grx1-roGFP2 signal superimposes with the spatially confined perinuclear TMRM signal and presents as a distinct rim around the nucleus because of the high nucleus to cytosol ratio in TSA cells. Scale bars: 50 μm. **(B)** Representative Western blot analysis to confirm enrichment of the respective roGFP2 reporters in cytosolic (c) and mitochondria (m) compartments. The mitochondrial compartment is characterized by proteins of the electron transport chain complexes I - IV (SDHB: Succinate dehydrogenase complex iron sulfur subunit B, MTCO1: Cytochrome c oxidase I, UQCRC2: ubiquinol-cytochrome c reductase core protein II, ATP5A: ATP synthase subunit alpha).

We first generated TSA cells stably expressing Grx1-roGFP2 and mito-Grx1-roGFP2 by co-transfection of linearized donor vectors encoding for the respective sensors and TALEN-expression vectors to verify compartment (cytosol vs. mitochondria)-specific sensor location. Sensor expressing cells were purified by selection with puromycin. Life imaging of TSA cells confirmed co-localization of GFP and the mitochondrial TMRM signals in mito-Grx1-roGFP2 cells, whereas Grx1-roGFP2 expressing cells showed a uniform cytosolic distribution of the GFP signal (Figure 1A – GFP panels). To further interrogate the cytosolic and mitochondrial localization of Grx1-roGFP2, we performed Western blots for GFP protein in cytosolic and mitochondrial protein fractions. For Grx1-roGFP2 cells, the GFP signal was detected primarily in the cytosolic fraction, whereas in mito-Grx1-roGFP2 enrichment of GFP in the mitochondrial fraction was observed (Figure 1B). Wild type control cells were negative for GFP.

### Generation of Functional GSH Reporter HES Cell Lines

**Figure 2:**
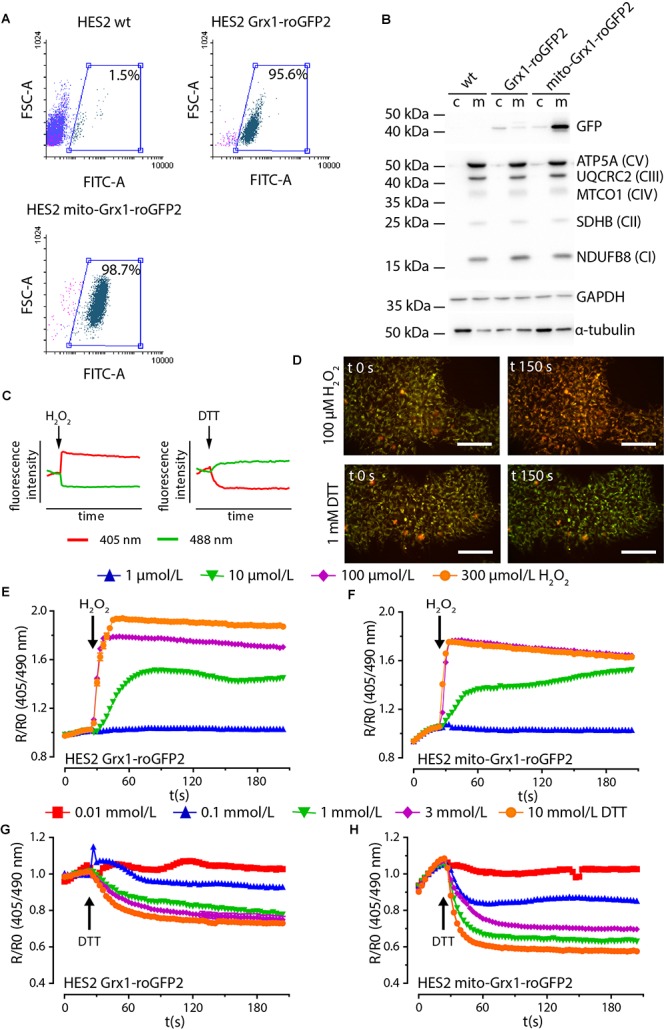
TALEN-modified HES2 express functional Grx1-roGFP2. **(A)** Flow cytometry analysis of GFP positive cells in genetically naïve (HES2 wt) and TALEN-modified Grx1-roGFP2 and mito-Grx1-roGFP2 HES2. **(B)** Representative Western blot analysis to confirm enrichment of the respective roGFP2 reporters in cytosolic (c) and mitochondria (m) compartments. The mitochondrial compartment is characterized by proteins of the electron transport chain complexes I - IV (NDUFB8: NADH: ubiquinone oxidoreductase subunit B8, SDHB: Succinate dehydrogenase complex iron sulfur subunit B, MTCO1: Cytochrome c oxidase I, UQCRC2: ubiquinol-cytochrome c reductase core protein II, ATP5A: ATP synthase subunit alpha). **(C)** Oxidation and reduction of roGFP2 results in an inversely correlated shift in fluorescence intensity under 408 and 488 nm excitation. **(D)** Fluorescence intensity shift in exemplary false colored images of HES2 mito-Grx1-roGFP2 cells at *t* = 0 s and *t* = 150 s upon oxidative (H_2_O_2_) and reductive (DTT) stimulation. Red: 400 nm excitation, green: 500 nm excitation, scale bars: 100 μm. **(E–H)** Change of roGFP2 fluorescence signal in HES2 Grx1-roGFP2 **(E,G)** and HES2 mito-Grx1-roGFP2 **(F,H)** as a function of time under oxidative (H_2_O_2_, E, F) and reductive (DTT, G, H) stimulation. H_2_O_2_ or DTT were added at 30 s. Grx1-roGFP2: *n* = 241 cells (300 μmol/L H_2_O_2_), *n* = 205 cells (100 μmol/L H_2_O_2_), *n* = 203 cells (10 μmol/L H_2_O_2_), *n* = 52 cells (1 μmol/L H_2_O_2_), *n* = 197 cells (10 mmol/L DTT), *n* = 264 cells (3 mmol/L DTT), *n* = 189 cells (1 mmol/L DTT), *n* = 58 cells (0.1 mmol/L DTT), *n* = 53 cells (0.01 mmol/L DTT); mito-Grx1-roGFP2: *n* = 182 cells (300 μmol/L H_2_O_2_), *n* = 301 cells (100 μmol/L H_2_O_2_), *n* = 305 cells (10 μmol/L H_2_O_2_), *n* = 57 cells (1 μmol/L H_2_O_2_), *n* = 193 cells (10 mmol/L DTT), *n* = 252 cells (3 mmol/L DTT), *n* = 274 cells (1 mmol/L DTT), *n* = 56 cells (0.1 mmol/L DTT), *n* = 57 cells (0.01 mmol/L DTT).

We next generated human embryonic stem cell lines (HES2 background) with stable expression of Grx1-roGFP2 and mito-Grx1-roGFP2 by TALEN-mediated integration. The two investigated clonal cell lines demonstrated a uniform GFP signal (>95%; Figure 2A and Supplementary Figure 1A) and expression of the canonical pluripotency markers Oct4, Nanog and Tra1-60 (Supplementary Figure 1B). Correct localization and expression of GSH sensors was confirmed by Western blot analysis (Figure 2B). Next, we performed live dual excitation, single emission microscopy to assess functionality of the expressed sensors. Grx1-roGFP2 emits light at 500–530 nm upon stimulation with either 405 nm or 488 nm wavelength. The intensity of emitted fluorescence of the Grx1-roGFP2 reporter changes with the cellular GSH/GSSG state in an inversely correlated manner (Figure 2C). Live cell ratiometric imaging allows for the quantification of the cellular GSH redox state in real time (Figure 2D). In both HES lines, Grx1-roGFP2-HES2 and mito-Grx1-roGFP2-HES2, the sensor reacted in a concentration dependent manner to oxidation by H_2_O_2_ with an increase in fluorescence ratio (R/R_0_) under 405 nm excitation over 488 nm excitation (Figure 2E,F). Reduction upon stimulation with increasing concentrations of DTT led to a concentration dependent decrease of the fluorescence ratio (Figure 2G, H). The maximally observed R/R_0_ upon oxidation with 300 μmol/L H_2_O_2_ was 1.9 ± 0.01 for Grx1-roGFP2-HES2 (*n* = 241 cells) and 1.8 ± 0.01 for mito-Grx1-roGFP2-HES2 (*n* = 182 cells). Upon reduction with 10 mmol/L DTT R/R_0_ decreased to 0.7 ± 0.007 in Grx1-roGFP2-HES2 (*n* = 197 cells) and 0.6 ± 0.003 in mito-Grx1-roGFP2-HES2 (*n* = 193 cells).

### GSH/GSSG Imaging in Grx1-roGFP2-HES2 Derived Cardiomyocytes

**Figure 3:**
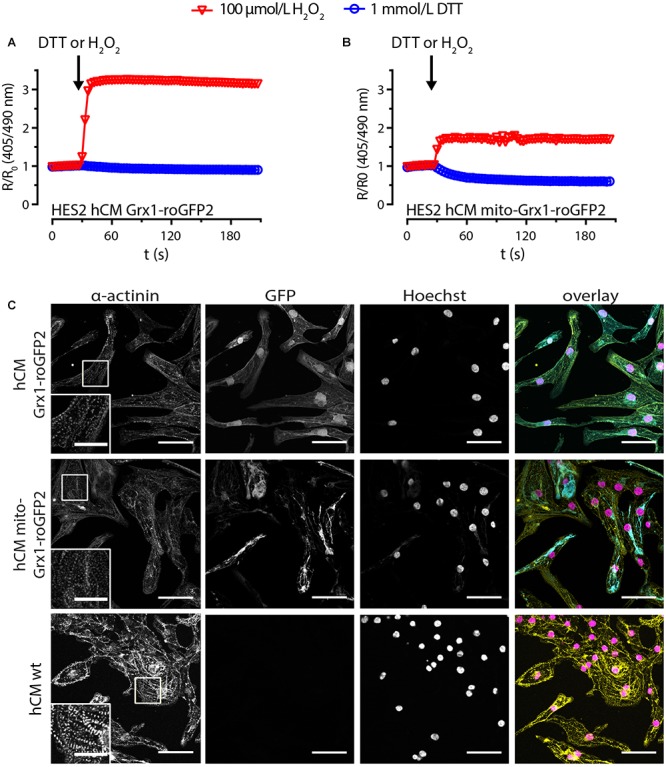
HES-derived cardiomyocytes express functional roGFP2 sensors, but show an impaired sarcomere phenotype. Change of roGFP2 fluorescence signal in hCM from HES2 Grx1-roGFP2 **(A)** and HES2 mito-Grx1-roGFP2 **(B)** as a function of time under oxidative (H_2_O_2_) and reductive (DTT) stimulation. Grx1-roGFP2: *n* = 176 cells (100 μmol/L H_2_O_2_), *n* = 170 cells (1 mmol/L DTT), mito-Grx1-roGFP2: *n* = 325 cells (100 μmol/L H_2_O_2_), *n* = 325 cells (1 mmol/L DTT). **(C)** Immunofluorescence analysis after staining for α-actinin and DNA (Hoechst); GFP: roGFP2 reporter signal. Scale bars: 50 μm. Insets: magnifications of α-actinin labeled sarcomere structures, scale bars: 20 μm.

We next subjected Grx1-roGFP2-HES2 and mito-Grx1-roGFP2-HES2 to a well-defined and highly robust directed cardiac differentiation protocol (Tiburcy et al., 2017). Flow cytometry analyses confirmed a yield of 80–95% α-actinin^+^ cardiomyocytes for both cell lines and no obvious differences to non-transgenic isogenic controls. Notably, all α-actinin^+^ Grx1-roGFP2 and mito-Grx1-roGFP2 cardiomyocytes (hCM) were also GFP^+^ by flow cytometry analysis (Supplementary Figure 2). Upon stimulation with 100 μmol/L H_2_O_2_ a maximal R/R_0_ of 3.3 ± 0.01 was observed in Grx1-roGFP2-hCM (*n* = 176 cells); a maximal R/R_0_ of 2.0 ± 0.1 was observed in mito-Grx1-roGFP2-hCM (*n* = 325 cells; Figure 3A,B). Maximal reduction upon 1 mmol/L DTT resulted in a R/R_0_ decrease to 0.9 ± 0.002 for Grx1-roGFP2-hCM (*n* = 170 cells) and 0.6 ± 0.005 for mito-Grx1-roGFP2-hCM (*n* = 325 cells). These findings were however, compromised by limited spontaneous beating activity (observed only in a small subsets of cardiomyocytes in 2 of 12 differentiations) and an obviously impaired sarcomerogenesis in the Grx1-roGFP2- and mito-Grx1-roGFP2-hCMs (Figure 3C, hCM wild type shown for comparison). To scrutinize whether the unanticipated dysfunction was due to the constitutive overexpression of Grx1-roGFP2 in our transgenic lines, we performed lentiviral transduction of differentiated, beating HES-derived hCM. Given the observation of similar developmental toxicity of the cytosolic and mitochondrial Grx1-roGFP2 variants, the greater R/R_0_ dynamic range in Grx1- versus mito-Grx1-roGFP2 cells, and our ultimate goal to establish proof-of-concept for in-tissue GSH/GSSG imaging, we decided to focus on the cytosolic Grx1-roGFP2 variant in the following experiments.

### Lentiviral Sensor Expression Does Not Impair Cardiomyocyte Function

**Figure 4:**
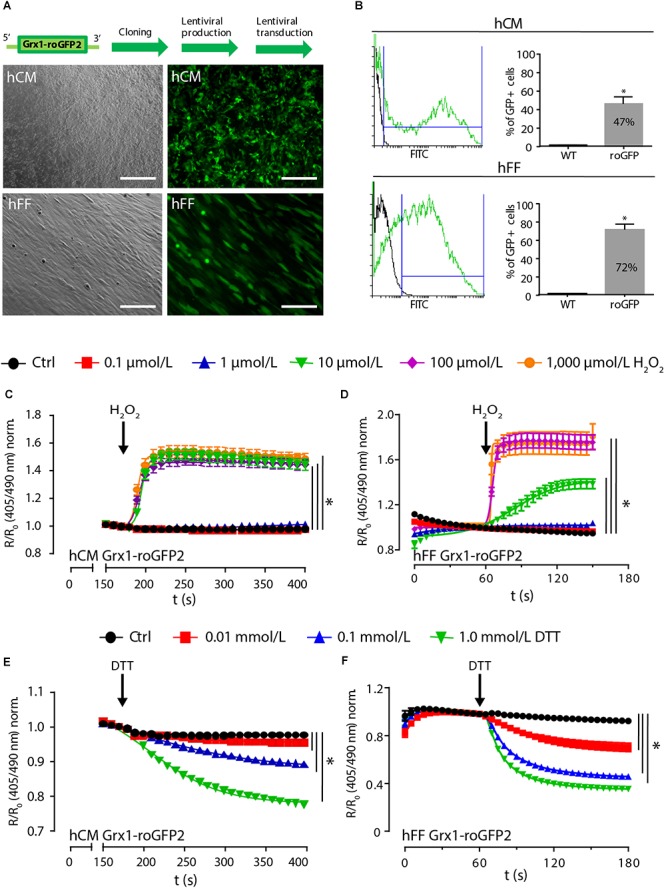
Lentiviral expression of Grx1-roGFP2 reports redox changes in differentiated cardiomyocytes and fibroblasts. **(A)** Brightfield (left) and GFP fluorescence (right) images after lentiviral transduction of hCM and hFF; scale bars: 200 μm. **(B)** Flow cytometry analysis of transduction efficiency in hCM (47 ± 7%, *n* = 7) and hFF (72 ± 6%, *n* = 8); ^∗^*p* < 0.05, unpaired, two-tailed Student’s *t*-test. Change of roGFP2 fluorescence signal in hCM **(C)** and hFF **(D)** as a function of time under oxidation by H_2_O_2_ at indicated concentrations (*n* = 46–71 [hCM]; *n* = 19–43 [hFF]). Change of roGFP2 fluorescence signal in hCM **(E)** and hFF **(F)** as a function of time under reduction by DTT at indicated concentrations (*n* = 46–85 [hCM]; *n* = 21–37 [hFF]).

Because fibroblasts play an essential role in natural and engineered cardiomyogenesis (Ieda et al., 2009; Tiburcy et al., 2017), we decided to lentivirally transduce differentiated HES2-derived hCM and human foreskin fibroblasts (hFF; Figure 4A) for subsequent use in the construction of contractile engineered human myocardium (EHM; Tiburcy et al., 2017). Transduction efficiency was 47 ± 7% for Grx1-roGFP2-hCM (*n* = 7) and 72 ± 6% for Grx1-roGFP2-hFF (*n* = 8; Figure 4B). In contrast to the observation in the directed differentiation of Grx1-roGFP2-HES2, lentivirally transduced hCM remained contractile with no obvious differences compared to non-transduced hCM. To examine whether GSH/GSSG imaging is possible, we exposed lentivirally (lenti) transduced Grx1-roGFP2-hCM (Figure 4C) and Grx1-roGFP2-hFF (Figure 4D) to increasing concentrations of H_2_O_2_ (0.1–1,000 μmol/L) and DTT (0.01–1 mmol/L). Lenti-Grx1-roGFP2-hCM presented an all-or-nothing response at ≥ 10 μmol/L H_2_O_2_ with a maximal R/R_0_ of 1.56 ± 0.04 at 100 μmol/L (*n* = 57; Figure 4C). In contrast, lenti-Grx1-roGFP2-hFF showed a concentration dependent increase of R/R_0_ with maximal oxidation at 100 μmol/L H_2_O_2_ (1.78 ± 0.07, *n* = 41; Figure 4D). Maximal reduction was observed at 1 mmol/L DTT with a R/R_0_ of 0.78 ± 0.01 (*n* = 58) and 0.36 ± 0.02 (*n* = 28) in lenti-Grx1-roGFP2-hCM (Figure 4E) and lenti-Grx1-roGFP2-hFF (Figure 4F), respectively. The estimated E_GSH_ was -289 ± 1 mV in Grx1-roGFP2-hCM and -269 ± 2 mV in Grx1-roGFP2-hFF (*p* < 0.05 by two-tailed, unpaired Student’s *t*-test). As the reaction of hFF to oxidation or reduction appeared to be faster than in hCM, we quantified the time to half maximal ratio (t_50_). In hCM, upon stimulation with 1,000 μmol/L H_2_O_2_, t_50_ was reached after 22 ± 2 ms (*n* = 39), while t_50_ in hFF was observed at 5 ± 0.3 ms (*n* = 41). Similarly, upon reduction with 1 mmol/L DTT, t_50_ was reached in 28 ± 1 ms in HFF (*n* = 28) and in 83 ± 3 ms in hCM (*n* = 58).

### Redox Imaging in Engineered Human Myocardium

**Figure 5:**
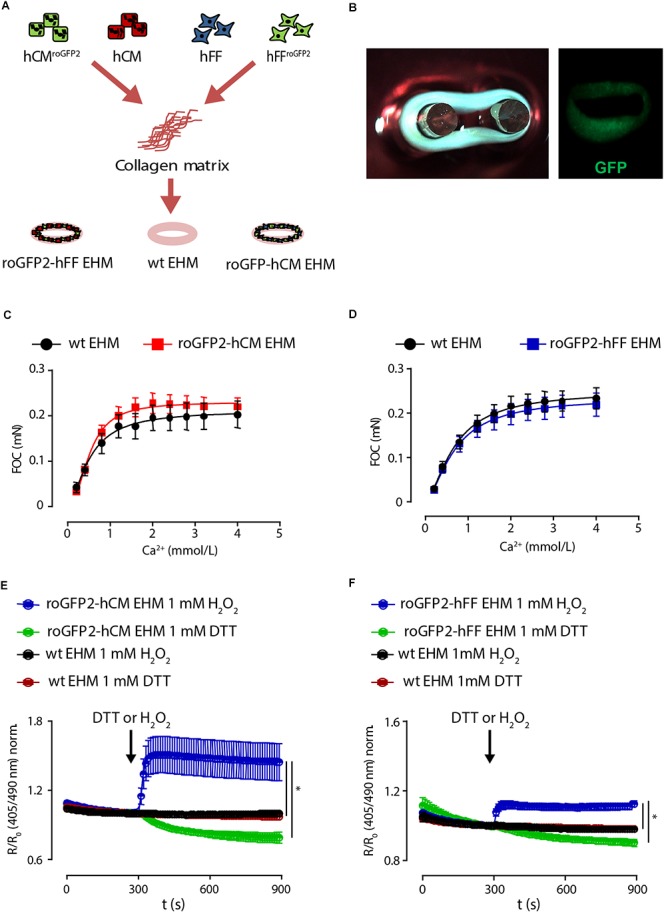
Cell type specific imaging of Grx1-roGFP2 in EHM. **(A)** EHM were constructed from defined mixtures of genetically naïve and Grx1-roGFP2 expressing hCM or hFF in a collagen hydrogel to create tissue for cell type specific redox potential imaging. **(B)** Photograph and fluorescence image of EHM expressing Grx1-roGFP2 (GFP). Analysis of force of contraction (FOC) under isometric conditions and electrical field stimulation (1.5 Hz); maximal inotropic capacity was evaluated under increasing extracellular calcium concentrations: **(C)** EHM composed of Grx1-roGFP2 and genetically naïve (wt: wild type) hCM with genetically naïve hFF (*n* = 17/33); **(D)** EHM composed of Grx1-roGFP2 and genetically naïve (wt: wild type) hFF with genetically naïve hCM (*n* = 36/42). Change of roGFP2 fluorescence signal in EHM with Grx1-roGFP2 expressing hCM **(E)** and hFF **(F)** as a function of time under oxidation by H_2_O_2_ (1 mmol/L) and reduction by DTT (1 mmol/L).

We finally tested whether lenti-Grx1-roGFP2-hCM and -hFF could be applied to perform cell-type specific in-tissue GSH/GSSG imaging in EHM. For this, we constructed EHM from defined mixtures of the Grx1-roGFP2-hCM (70%) and -hFF (30%) as recently described (Figure 5A,B; Tiburcy et al., 2017). To first test whether the Grx1-roGFP2 would disturb function, we performed force of contraction (FOC) analyses under isometric conditions and increasing calcium concentrations. These analyses confirmed that expression of the Grx1-roGFP2 reporter *per se* in differentiated hCM (Figure 5C) or hFF (Figure 5D) does not impair heart muscle function. We next tested whether cell type specific glutathione redox state can be imaged in EHM after exposure to maximally oxidizing concentrations of H_2_O_2_ (1 mmol/L) and maximally reducing concentrations of DTT (1 mmol/L). These experiments demonstrated a more pronounced fluorescence signal response to H_2_O_2_ and DTT in EHM comprised of lenti-Grx1-roGFP2-hCM and genetically naïve hFF (Figure 5E) compared to EHM comprised of genetically naïve hCM and lenti-Grx1-roGFP2-hFF (Figure 5F). This finding may be explained by the smaller cell body of hFF and the lower signal to noise (S/N) ratio in hFF-roGFP2 compared to the hCM-roGFP2. The E_GSH_ was -284 ± 4 mV for lenti-Grx-roGFP2-hCM containing EHM and -297 ± 34 mV for lenti-Grx-roGFP2-hFF containing EHM (*n* = 5 tissues/group).

## Discussion

Reactive oxygen species contribute to a variety of cardiac functions by their fundamental role in physiological signaling and pathological processes in the heart. The ROS production machinery and how ROS target individual proteins of the contractile machinery has been intensively investigated (Shao et al., 2012; Zhang et al., 2013; Bertero and Maack, 2018). However, knowledge on spatiotemporal activity of ROS in distinct cell species of the heart and their cellular compartments remains incomplete. Here we sought to develop a human heart muscle model for cell type-specific, in-tissue glutathione redox potential imaging via a recently developed protein sensor, namely Grx1-roGFP2 (Gutscher et al., 2008). We tested two different approaches for the generation of Grx1-roGFP2 expressing cardiomyocytes: (1) TALEN-mediated genome integration in human embryonic stem cells for subsequent cardiac differentiation and (2) lentiviral transduction of differentiated cardiomyocytes. Whilst TALEN-mediated integration of Grx1-roGFP2 for cytosolic and mitochondrial sensing of GSH/GSSG ratio was possible, we observed severely compromised sarcomerogenesis leading to contractile dysfunction in the two investigated Grx1-roGFP2 models. This may stem from the observed multiple random integrations, despite initially targeting the AAVS1 locus. However, the finding that sarcomerogenesis was similarly impaired in the Grx1- and mito-Grx1-roGFP2 lines, which exhibit different transgene integration sites, whilst lentiviral transduction of already differentiated cardiomyocytes was not toxic, argues for developmental toxicity of the roGFP2 reporter. One possible mechanism could be ROS buffering and thus impaired ROS signaling during early stages of cardiomyogenesis. Further investigations, including the screening for additional lines with random and AAVS1 targeted transgene integrations, will be required to clarify the underlying mechanism.

The finding of non-impaired cardiomyocyte function after lentiviral transduction is in line with the observation that transgenic mice with Grx1-roGFP2 expression under the control of the cardiomyocyte restricted MYH6-promotor do not show an obvious phenotype (Swain et al., 2016). With the transgenic expression of lenti-Grx1-roGFP2 in human cardiomyocytes and fibroblasts, it became feasible to construct engineered human myocardium (EHM) for in-tissue GSG/GSSG imaging. Importantly, the unimpaired contractile performance of EHM is a strong indicator for undisturbed sarcomerogenesis in EHM. This notion is supported by a number of studies showing the high sensitivity of the EHM model to pick up mechanisms underlying contractile dysfunction in patients with cardiomyopathy, such as in Duchenne Muscular Dystrophy (Long et al., 2018) and dilated cardiomyopathy (Streckfuss-Bömeke et al., 2017). The finding that Grx1-roGFP2 expressing fibroblasts in EHM do not compromise its contractile function is another important observation, given the requirement for proper fibroblast function in *bona fide* and engineered cardiomyogenesis (Ieda et al., 2009; Tiburcy et al., 2017).

The differences in hCM-roGFP2 versus hFF-roGFP2 R/R_0_ signal dynamic range in EHM (Figure 5E,F) may be explained by differences in cell composition (hCM > hFF) and cell volume (hCM > hFF) as well as differences in cell type-specific roGFP2 protein translation and redox state. Similarly, the smaller R/R_0_ signal dynamic range of the hFF-roGFP2-EHM (Figure 5F) versus hFF-roGFP2-monolayer (Figure 4D and F) signal may stem from the smaller hFF cell body in EHM versus monolayer culture. The differences in hFF size are a reflection of differences in fibroblast biology in monolayer and EHM culture, with a dominant “stressed” myofibroblast phenotype in monolayer culture and a less “stressed” fibrocyte phenotype in EHM culture.

The determination of the maximal oxidative and reductive states in cells allows for the calculation of the glutathione redox potential (E_GSH_; Meyer and Dick, 2010). Here, we identify apparent differences in E_GSH_ in cardiomyocytes and fibroblasts (-289 ± 1 mV vs. -269 ± 2 mV), supporting the notion that cell specific analyses will be key to understand the pathophysiological relevance of ROS. Interestingly, the cardiomyocyte and fibroblast E_GSH_ in EHM appeared to not differ, suggesting either a milieu effect or differences in E_GSH_ in the more matured cells in three-dimensional EHM versus monolayer cultures (Tiburcy et al., 2017).

By the use of the glutathione sensor (Grx1-roGFP2) we were naturally limited in our investigations to only one ROS-mechanism, namely glutathione oxidation/reduction. The use of additional ROS sensors as well as a combination of transgenic reporters and ROS-producing enzymes in a cell and compartment specific manner may be highly informative to decipher ROS signaling. In this context, we interpret our study as a first step toward this direction with proof-of-concept for the feasibility of in-human-heart-muscle GSH/GSSG imaging. Further studies will be required to make full use of the Grx1-roGFP2-reporter EHM model, which will include investigations of the consequences of specific pharmacological (e.g., simulated neurohumoral overstimulation), biophysical (e.g., controlled electromechanical stimulation), or disease (e.g., hypoxia or ischemia/reoxygenation) stimuli for the ROS-mediated control of heart muscle function.

## Data Availability

All datasets generated for this study are included in the manuscript and/or the Supplementary Files.

## Author Contributions

IT, EH, PS, MM, and WHZ contributed to the conception and design of the study. IT, EH, and EL performed the experiments. VN, DMK, and IB supported the roGFP2 sensor imaging experiments. IT, EH, PS, and WHZ drafted the manuscript. All the authors contributed to manuscript revision, read, and approved the submitted version.

## Conflict of Interest Statement

The authors declare that the research was conducted in the absence of any commercial or financial relationships that could be construed as a potential conflict of interest.
