# Supplementary material for: Optogenetic Monitoring of the Glutathione Redox State in Engineered Human Myocardium
Source: Front Physiol. 2019 Apr 4;10:272. doi: 10.3389/fphys.2019.00272 (PMC6460052; doi:10.3389/fphys.2019.00272)
Supplement: Supplementary file 1 [file Data_Sheet_1.PDF]

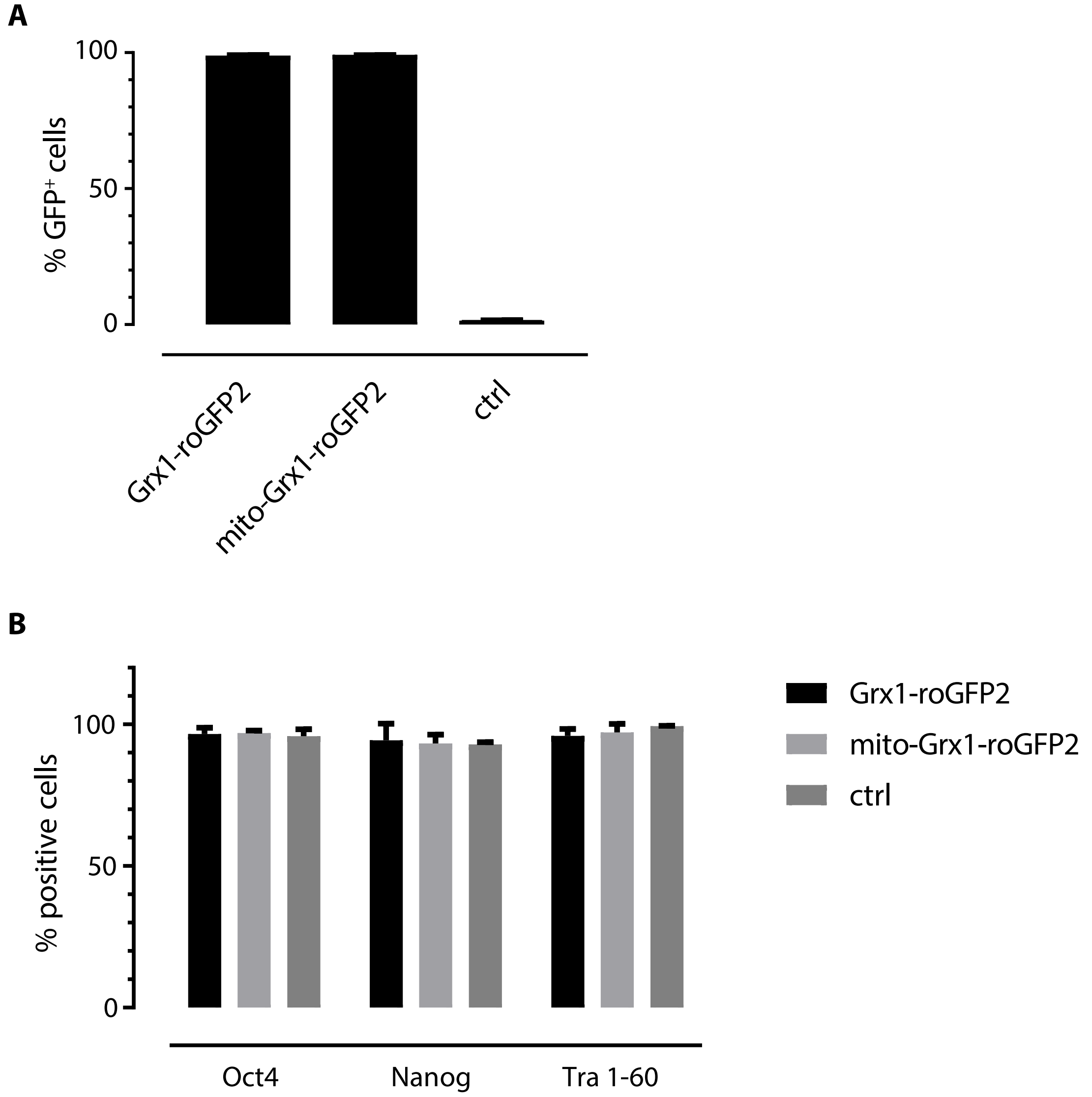

Supplemental figure 1. Reporter expression and pluripotency analysis in HES2 lines.
(**A**) Live cell flow cytometry analysis demonstrated uniform GFP expression in both reporter HES lines (cells from n = 3 independent passages / cell line). (**B**) Flow cytometry analysis of Oct4, Nanog, and Tra 1-60 demonstrates that pluripotency marker expression is unimpaired in Grx1-roGFP2 and mito-Grx1-roGFP2 HES lines (cells from n = 3 independent passages / cell line).

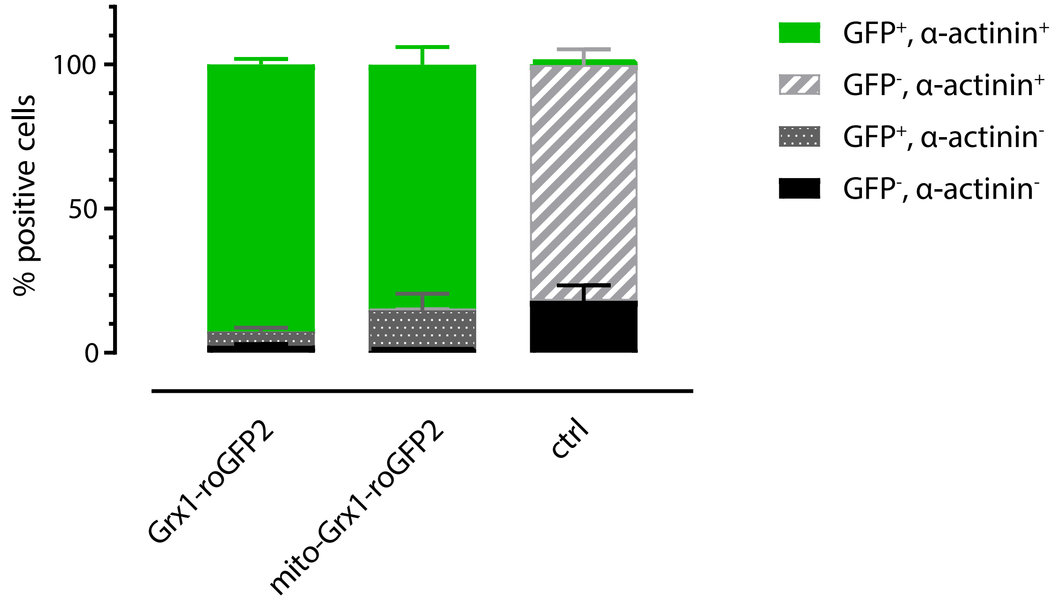

Supplemental figure 2. Flow cytometry analysis of roGFP2 reporter expression in hCM.
Flow cytometry analysis for GFP and α-actinin in sensor expressing hCM and a non-transgenic control line (n = 3 differentiations / cell line).
